# Housing instability, health, and substance use among adults over age 50 living in New York City

**DOI:** 10.3389/fpubh.2025.1725374

**Published:** 2026-01-20

**Authors:** H. Shellae Versey, Michelle Dougherty, Christina Mair

**Affiliations:** 1 Department of Psychology, Fordham University, New York, NY, United States; 2 Department of Behavioral and Community Health Sciences, University of Pittsburgh School of Public Health, Pittsburgh, PA, United States

**Keywords:** health, housing insecurity, housing instability, neighborhood, social contexts, NORCs, older adults, substance use

## Abstract

**Introduction:**

As cities become increasingly expensive, it is unclear how rising housing costs impact older adults’ health and the ability to age-in-place.

**Methods:**

We examined key indicators of housing instability – concerns about paying rent/mortgage on time and number of moves in the past 5 years – in relation to health and substance use among a sample of adults over age 50 with a history of cannabis use living in naturally occurring retirement communities (NORCs) in New York City.

**Results:**

Bivariate analyses and logistic regression models indicate a significant relationship between housing instability and health, such that concerns about paying rent or mortgage were associated with worse self-reported health, and higher tobacco and other substance use. Frequent moves were associated with poorer health and simultaneous alcohol and cannabis co-use in bivariate analyses. Overall, housing instability was consistently associated with worse health and higher rates of substance use, particularly tobacco and other substances, among those who also use cannabis.

**Discussion:**

Findings suggest that NORC-dwelling does not fully alleviate housing costs or concerns in densely populated, high-cost cities. Specifically for older adults with a history of cannabis use, housing concerns may be an important correlate of substance use. As NORCs continue to integrate a range of health and social services offerings to achieve aging-in-place goals, substance use and financial literacy programs could be useful additions. Broadly, correlations between substance use and housing stress among older adults warrants additional attention.

## Introduction

Housing instability among older adults is rising and housing-related issues (e.g., affordability) are associated with poorer health (1, 2). According to reports, older adults are one of the fastest growing groups affected by housing instability (3). Characterized by challenges in maintaining housing, instability includes having trouble paying rent or mortgage, overcrowding, moving frequently, and spending most of household income on housing (4, 5). In the U. S., an estimated 30% of households experience some form of housing instability annually (6). Approximately 10% of U. S. older adults (aged 65 and older) have annual incomes below the federal poverty level, and nearly one-third of older adult households in the U. S. spend more than 30% of their incomes on housing costs (7). Lower-income older adults are also more likely to rent than own a home compared higher-income households (8, 9). Consequently, older adults with limited incomes may be at increased risk for housing instability given rising rents, existing poorer health, lower housing quality, social isolation, and financial victimization (10), yet most previous research has focused on young adults and children (10–13). Given these increased risks, older adults are a neglected demographic in the housing instability literature (8).

Housing instability is primarily driven by policies and structural factors that make maintaining stable and permanent housing difficult. Recent challenges resulting from the recession followed by the COVID-19 pandemic have contributed to a tight housing market, where affordability is at a 30-year low and housing costs have risen exponentially (14). Previous research indicates that housing-related stress may impact health, psychological well-being, and behavioral coping (e.g., substance use) particularly for lower-income residents and minoritized older adults (15–17). However, while the extant literature has suggested a general link between housing and health, there is less known about behavioral correlates, such as substance use, that may accompany housing instability and stress among older adults (18). In addition, the few studies that have examined these relationships have focused on homelessness, rather than housing instability among already housed adults (19–21). The association between housing-related stress and substance use is complex and likely bidirectional, since substance use can be both a driver and consequence of housing instability. We hypothesize that substance use is correlated with housing stress, aligned with a general ecosocial and social causation framework (22, 23).

To better understand relationships between housing instability and substance use, we examine self-reports of housing concerns and the number of residential moves in relation to self-rated health and substance use (e.g., cannabis use, alcohol, and tobacco) in a community-dwelling, urban sample of adults over age 50 with a history of cannabis use. We purposively sampled older adults living in naturally occurring retirement communities (NORCs) in New York City (NYC), since previous research suggests that NORCs may offer housing stability and promote aging-in-place in areas where housing demands are high (24–26). Understanding how (and if) NORCs – traditionally defined as relatively stable, residential communities that house a large proportion of older adults – fully support aging-in-place and contribute to health may inform future research and housing models.

## Methods

In 2022, we recruited and administered a web-based survey to a diverse sample of NORC-dwelling older adults with a history of cannabis use across six neighborhoods in the Manhattan and Bronx boroughs of NYC. Due to the focus on understanding the impact of COVID-related housing stability and new cannabis legalization, study recruitment focused on people who have used cannabis at least once in their lifetime. Sampling occurred at two levels of analysis: neighborhoods and NORC sites. First, neighborhoods within two boroughs (Manhattan and the Bronx) were chosen based on the research team’s preexisting relationships with community partners. We focused on six neighborhoods corresponding to 2020 Neighborhood Tabulation Areas: three in the Bronx (Co-op City, Pelham Parkway, Parkchester), and three in Manhattan (Lower East Side, Harlem/Morningside Heights, and the Upper West Side) (27). We chose these neighborhoods in part for their racial and ethnic diversity; for example, in Parkchester, 25.4% of residents identify as Asian, 35.1% as Black, 33.0% as Hispanic/Latiné, and 2.7% as White, while in the Lower East Side, 25.0% of residents identify as Asian, 9.2% as Black, 30.7% as Hispanic/Latiné, and 31.2% as White (27).

Second, NORC sites were identified using a list and map of NORC-defined buildings and NYC Department for the Aging NORC program sites (where supportive services are offered) generated by the city of New York (28). NORC sites constitute unified apartment buildings or complexes designated by the NYC Department for the Aging (DFTA). As of 2024, New York City had at least 60 NORCS that have a social service provider.^1^ At the time of recruitment, there were five NORCs in the Bronx and 17 in Manhattan. The recruitment team contacted all sites within selected neighborhoods and, for sites where building managers gave permission, held in-person recruitment events and posted flyers.

### Eligibility

Eligibility for study participation was open to NORC-dwelling older adults aged 50 and older living in the Manhattan and Bronx boroughs of NYC (though not a requirement) who had used cannabis at least once in their lifetimes [“Have you ever used marijuana (e.g., cannabis, weed, hash, pot, edibles)?”]. Targeting recruitment to adults who lived in NORCs and had used cannabis at least once in their lifetimes was key to understanding the primary research question. At the time of data collection, recreational cannabis use among adults in New York had been legalized for nearly a year (since March 31, 2021) and cannabis was available through dispensaries, though licensed recreational stores had not yet opened (29).

The survey was offered in both English and Spanish; however, all respondents chose to take the English version. A $15 gift card was provided to all participants who completed the survey. The Institutional Review Board at Fordham University and the University of Pittsburgh reviewed and approved this study prior to data collection.

### Participant recruitment

The recruitment team worked closely with on-site community partners, anticipating difficulty in recruitment given the stigma associated with cannabis use and potential medical/research mistrust (30–32). The team also used a variety of recruitment strategies, including posting flyers at nearby businesses in NORC-designated areas (including corner stores, dispensaries, and smoke/vape shops) and conducting in-person, on-the-street recruitment. Participants could access the survey via a QR code on the study flyer or take a survey in person. For in-person recruitment events and on-the-street recruitment, research team members gave participants an iPad on which to take the survey, offering instructions and answering questions about the study as needed.

To maximize recruitment efforts, we conducted snowball sampling by offering a referral code to all participants who completed the survey, awarding $5 per referral (up to $15 per person in referral bonuses). Though recruitment targeted adults living in NORCs in Manhattan and the Bronx, we did not exclude participants from other parts of the city (or living temporarily in the Bronx) who completed the survey. Our recruitment team consisted of three recruiters, two Black/African American women and one white woman ranging in age from 20 to 40 years old, similar to the demographics of those we recruited, though younger in age.

Eligibility was assessed using screening questions at the beginning of the survey to confirm that participants consented and met the inclusion criteria (age 50 or older and had ever used cannabis in their lifetime). Participants were included regardless of how long they had lived in their current neighborhood. Of the 255 people that initiated the survey, 182 met eligibility inclusion criteria and consented to continue with the survey. Of these, 159 had complete data for key outcomes and covariates of interest. In some of our analyses we used this sample of older adults with lifetime cannabis use (*N =* 159), while in other analyses we restricted our sample further to those with past-year cannabis use (*N =* 123), as described further below.

### Measures

In developing the survey, we sought feedback from community partners (e.g., social service coordinators and building managers) on recruitment materials and terminology used for survey items. We incorporated this feedback into a finalized survey, which included questions on cannabis use, general substance use, neighborhood contexts, health, and key demographics.

#### Demographics

For key demographics, we measured age as a continuous indicator. We also assessed race and ethnicity (White non-Hispanic/Latiné vs. Black/ Hispanic/Latiné /Other races/ethnicities), gender identity (Male, Female, and Other identities), sexual orientation (Heterosexual vs. Minoritized Sexual Orientations), and relationship status (married or living with a partner vs. not married/living with partner, which included divorced, widowed, or never married). Other socioeconomic indicators included employment status (full-time, part-time, or not employed, which included those who were unemployed or retired) and highest level of education completed (high school or less vs. some college or college graduate). Participants reported their yearly household income by selecting options presented in increments of $10,000, which we then grouped into three categories based on the distribution reported: <20,000 (1), $20,000–$60,000 (2) and >60,000 (3).

#### Self-rated health

Self-rated health was measured with a single item. We asked participants, “How is your health in general?” and grouped response options into the following two categories: Poor/Very Poor/Fair vs. Good/Excellent.

#### Cannabis use frequency

To assess frequency of cannabis use we used a question from the Cannabis Use Disorders Identification Test (CUDIT) scale (“How often do you use marijuana?”) (33). We also asked participants when they last used cannabis. We then created a dichotomous indicator equal to one if participants had used weekly or daily within the past year and zero otherwise.

#### Cannabis-alcohol simultaneous use

To assess the frequency of cannabis and alcohol use simultaneously, we asked, “In the past year, how often did you use alcohol and marijuana (or marijuana products) at the same time?” We recoded response options to create a dichotomous variable to indicate whether participants had used cannabis and alcohol simultaneously within the past year (yes = 1) or not (no = 0).

#### Past-year use of other substances

To assess the use of other substances (not cannabis), we asked participants to indicate which of the following substances they had used in the past year: alcohol, tobacco, prescription pain killers (e.g., oxycodone, Vicodin), downers (e.g., Valium, Ativan, Xanax), heroin, cocaine, crack, poppers/nitrites, methamphetamines (e.g., crystal, tine, meth, speed) and psychedelics (e.g., acid, LSD, mushrooms, PCP). Participants could select multiple responses. In our bivariate and multivariate analyses, we used separate indicators for past- year 1) alcohol use, 2) tobacco use, and 3) all other substances.

#### Housing instability

We used two measures of housing instability. First, we asked participants to indicate if they were currently confident in their ability to pay their rent or mortgage on time, which we reverse coded for analyses to indicate if they were concerned with rent or mortgage payments. We also asked participants how many times they had moved in the past 5 years (e.g., Never, Once, 2–5 times, 5–10 times) and, based on the response distribution, we created a dichotomous indicator of whether participants had moved twice or more in the past 5 years as a proxy for housing instability. Given the urban context where one residential move may not sufficiently indicate instability, a cutoff of twice in a five- year period was used.

### Analysis

To describe the sample, we calculated means and standard deviations for continuous variables and frequencies for categorical variables. We analyzed bivariate differences in rent/mortgage concerns, history of residential mobility, and health. Using a multivariable regression model, we assessed which factors were associated with self-rated health, substance use, and polysubstance use (e.g., cannabis, tobacco, and alcohol co-use).

Our independent variables of interest were whether participants reported: (1) being concerned with paying rent or mortgage, and (2) moving twice or more in the past 5 years. Among adults with lifetime cannabis use (*N =* 159), we assessed whether these two independent variables of interest were associated with differences in self-rated physical health, using cannabis weekly or daily within the past year, and cannabis-alcohol simultaneous use within the past year. Because we were also interested in potential polysubstance use, we examined variation in past year use of: (1) alcohol, (2) tobacco, and (3) other substances (methamphetamines, cocaine, etc., as described above) among those who used cannabis within the past year (*N =* 123).

To assess the association between proxies of housing instability and outcomes of interest, we first conducted bivariate analyses. For outcomes that had significant bivariate relationships with housing instability measures, we used multivariable logistic regression to further test these relationships while controlling for demographic and economic factors. Given the exploratory nature of our study, we did not adjust for multiple comparisons (34).

## Results

Findings are based on a sample of community-dwelling adults with a history of cannabis use (*N =* 159) recruited from NORCs in six NYC neighborhoods, three in the Bronx and three in Manhattan. On average, participants were 60 years of age, and most identified as Black, Hispanic/Latiné, or multiracial (71.1%). The majority of respondents were men (55.3%) and identified as heterosexual (78.6%). Regarding key housing indicators, 32% indicated that they were currently concerned with paying rent or mortgage on time, and 20% reported having moved at least once in the past 5 years. Nearly 11% of respondents reported ever being evicted. Overall, nearly half (45.9%) of adults surveyed reported using cannabis weekly or more frequently and over half (56.6%) reported being in “good” or “excellent” health (see Table 1). At the same time, 48% of participants reported using substances other than alcohol or tobacco in the past year, including prescription pain killers (30.2%), downers (30.8%), heroin (23.9%), and cocaine (25.8%).

**Table 1:** Characteristics of older adults with lifetime cannabis use (*N =* 159).

Age (years), mean (SD)	60.0 (7.5)
Race and Ethnicity, *n* (%)	
Black, Latine, Other/Multiracial	113 (71.1%)
White, non-Latine	46 (28.9%)
Gender identity, *n* (%)	
Male	88 (55.3%)
Female	57 (35.8%)
Other	14 (8.8%)
Sexual orientation, *n* (%)	
Heterosexual	125 (78.6%)
Minoritized sexual orientation	34 (21.4%)
Relationship status, *n* (%)	
Married/living w partner	49 (31.0%)
Single	109 (69.0%)
Employment, *n* (%)	
Full time	47 (29.6%)
Part time	39 (24.5%)
Not employed	73 (45.9%)
Highest level of education, *n* (%)	
High school or less	82 (51.6%)
Some college or college graduate	77 (48.4%)
Income, *n* (%)	
<$20 k	62 (39.0%)
$20 k–60 k	62 (39.0%)
>60 k	35 (22.0%)
Moved twice or more in past 5 years, *n* (%)	32 (20.1%)
Concerned with paying rent/mortgage, *n* (%)	51 (32.1%)
Ever been evicted, *n* (%)	17 (10.8%)
Self-rated health status	
Fair/Poor/Very Poor	69 (43.4%)
Good/Excellent	90 (56.6%)
Current cannabis use weekly or daily	73 (45.9%)
Cannabis alcohol simultaneous in past year, *n* (%)	101 (63.5%)
Used alcohol in past year, *n* (%)	115 (72.3%)
Used tobacco in past year, *n* (%)	68 (42.8%)
Used other substances in past year, *n* (%), including:	77 (48.4%)
Prescription pain killers, *n* (%)	48 (30.2%)
Downers, *n* (%)	48 (30.8%)
Heroin, *n* (%)	38 (23.9%)
Cocaine, *n* (%)	40 (25.8%)
Crack, *n* (%)	43 (27.2%)
Poppers/nitrates, *n* (%)	34 (21.7%)
Methamphetamines, *n* (%)	29 (18.4%)
Psychedelics, *n* (%)	37 (23.4%)

Bivariate comparisons indicate a significant relationship between concerns about paying rent or mortgage, self-rated health, and cannabis-alcohol simultaneous use among those with lifetime cannabis use (*N =* 159). Concerns about paying rent or mortgage were related to poorer health. Among participants reporting cannabis use in the past year (*N =* 123), concerns about housing were associated with tobacco use and other substance use in the past year (see Table 2). Similarly, among those reporting cannabis use in the past year, moving twice or more (compared to once or less) was associated with increased tobacco use and other substance use in the past year. Frequent moves were correlated with cannabis and alcohol use simultaneously and report poorer health (see Table 3).

**Table 2:** Bivariate analysis of health status and substance use measures by concerns about paying rent/mortgage.

	Concern with paying rent or mortgage Among those with lifetime cannabis use (*N* = 159)		
	No (*N =* 108)	Yes (*N =* 51)	Test
Self-rated health status: Good/ Excellent^a^	78 (72.2%)	12 (23.5%)	<0.001
Current cannabis use weekly or daily	50 (46.3%)	23 (45.1%)	0.887
Cannabis alcohol simultaneous in past year	57 (52.8%)	44 (86.3%)	<0.001

	Among those with past year cannabis use (*N* = 123)		
	No (*N =* 89)	Yes (*N =* 34)	Test
Used alcohol in past year	68 (76.4%)	26 (76.5%)	0.994
Used tobacco in past year	26 (29.2%)	25 (73.5%)	<0.001
Used other substances in past year	28 (31.5%)	26 (76.5%)	<0.001

**Table 3:** Bivariate analysis of health status and substance use by number of times moved in past 5 years.

	Times moved in past 5 yearsAmong those with lifetime cannabis use (*N* = 159)		
	Once or less (*N =* 127)	Twice or more (*N =* 32)	Test
Self-rated health status: Good/ Excellent^a^	84 (66.1%)	6 (18.8%)	<0.001
Current cannabis use weekly or daily	61 (48.0%)	12 (37.5%)	0.285
Cannabis alcohol simultaneous in past year	72 (56.7%)	29 (90.6%)	<0.001

	Among those with past year cannabis (*N* = 123)		
	Once or less (*N =* 102)	Twice or more (*N =* 21)	Test
Used alcohol in past year	76 (74.5%)	18 (85.7%)	0.271
Used tobacco in past year	34 (33.3%)	17 (81.0%)	<0.001
Used other substances in past year	38 (37.3%)	16 (76.2%)	0.001

^a^Participants categorized as “Yes” refers to endorsing either “Good” or “Excellent” response option in replying to the question, “How is your health in general?” These two categories were grouped together. Response categories Poor/Very Poor/Fair were grouped together and categorized as “No.”

In adjusted models after controlling for demographic factors, older adults with lifetime cannabis use who expressed concern about paying rent or mortgage reported poorer health (AOR = 0.38, 95% CI: 0.15–0.99). Moving frequently (twice or more) also trends towards poorer health. Among participants with past-year cannabis use, both housing instability indicators – concern about paying rent/mortgage and frequent mobility – were associated with increased odds of past-year tobacco use, where concerns with paying rent/mortgage indicated a fivefold higher odds of tobacco use (AOR = 5.31, 95% CI: 1.64–17.24). Concern about housing cost was also significantly associated with other substance use (AOR = 3.77, 95% CI: 1.23–11.59). Frequent mobility (moving twice or more in the past 5 years) was associated with tobacco use (AOR = 4.08, 95% CI: 1.04–15.99), but not with use of other substances (see Tables 4, 5). Additionally, White participants had significantly higher odds of other substance use compared to adults identifying as non-White or Hispanic/Latiné (AOR = 2.96, 95% CI: 1.05–8.40).

**Table 4:** Multivariable logistic regression results of health status and simultaneous alcohol-cannabis use among older adults with lifetime cannabis use (*N =* 159).

	Good/excellent health status			Simultaneous alcohol-cannabis use		
	AOR	(95% CI)	*p*-value	AOR	(95% CI)	*p*-value
Concerned with paying rent/mortgage (reference = No concern)	0.379*	(0.145, 0.989)	0.047	2.861	(0.990, 8.264)	0.052
Moved twice or more in past 5 years (reference = Once or less)	0.379	(0.113, 1.277)	0.118	3.398	(0.830, 13.917)	0.089
Age (years)	1.002	(0.944, 1.064)	0.938	0.997	(0.946, 1.052)	0.925
White Non-Latine Race/Ethnicity (reference = Non-White or Latine)	0.977	(0.372, 2.567)	0.963	0.549	(0.227, 1.331)	0.185
Gender identity (reference = Male)						
Female	1.572	(0.646, 3.821)	0.319	1.416	(0.641, 3.132)	0.390
Other	0.366	(0.069, 1.949)	0.239	0.857	(0.169, 4.338)	0.852
Minoritized sexual orientation (reference = Heterosexual)	0.744	(0.242, 2.284)	0.605	1.611	(0.493, 5.263)	0.430
Income (reference = <$20 k)						
$20 k-60 k	4.548*	(1.612, 12.83)	0.004	0.520	(0.185, 1.462)	0.215
>60 k	6.488*	(1.910, 22.042)	0.003	0.576	(0.187, 1.776)	0.337

AOR, Adjusted Odds Ratio; CI, Confidence Interval. *p < 0.05.

**Table 5:** Multivariable logistic regression results of past year tobacco and other substance use among older adults with past year cannabis use (*N =* 123).

	Past year tobacco use			Past year other substance use		
	AOR	(95% CI)	*p*-value	AOR	(95% CI)	*p*-value
Concerned with paying rent/mortgage (reference = No concern)	5.314*	(1.638, 17.236)	0.005	3.773*	(1.228, 11.590)	0.020
Moved twice or more in past 5 years (reference = Once or less)	4.079*	(1.041, 15.990)	0.044	1.637	(0.437, 6.134)	0.465
Age (years)	0.969	(0.908, 1.034)	0.336	1.029	(0.963, 1.099)	0.399
White Non-Latine Race/Ethnicity (reference = Non-White or Latine)	0.582	(0.199, 1.704)	0.324	2.963*	(1.045, 8.397)	0.041
Gender identity (reference = Male)						
Female	1.068	(0.422, 2.702)	0.890	1.763	(0.702, 4.43)	0.228
Other	0.278	(0.038, 2.023)	0.206	1.240	(0.191, 8.074)	0.822
Minoritized sexual orientation (reference = Heterosexual)	1.528	(0.404, 5.774)	0.532	2.261	(0.580, 8.818)	0.240
Income (reference = <$20 k)						
$20 k-60 k	0.404	(0.119, 1.372)	0.146	0.507	(0.150, 1.716)	0.275
>60 k	1.304	(0.359, 4.742)	0.687	0.752	(0.206, 2.739)	0.666

AOR, Adjusted Odds Ratio; CI, Confidence Interval. *p <0.05.

## Discussion

Housing instability—measured by concern about paying rent/mortgage and frequent residential mobility—was consistently associated with worse self-rated health and higher rates of substance use, particularly tobacco, among adults over age 50 living in New York City NORCs who also report cannabis use. Specifically, higher mobility (i.e., those who moved two or more times in the past 5 years) was significantly related to poorer self-rated health and higher rates of polysubstance use—including simultaneous cannabis and alcohol use, tobacco use, and other substances.

Interestingly, though our sample mostly identified as Black and Hispanic/Latiné, White, non- Hispanic/Latiné residents reported higher odds of other substance use—highlighting the need to tailor potential housing-substance use interventions across ethnic-racial lines. Our results also support previous research that income does not fully buffer against substance co-use. This effect may be due to the overall demographics of our sample (i.e., mostly lower income); therefore, current income may be less useful as an indicator of current SES. These findings suggest that housing instability is an important, yet understudied social determinant of both health and substance use behaviors among older adults.

### Implications for older adults and NORCs

NORCs are informal aging-in-place models intended to support adults growing older in their existing homes and communities. However, our findings indicate that concerns about housing costs still affect NORC-dwelling residents, potentially underestimating the significant impact of economic insecurity experienced among adults living in high-cost cities. Practical implications of this work suggest that NORC-dwelling residents, particularly those with a history of cannabis use, may benefit from additional direct or community-based supports aimed at reducing housing instability. Though still evolving, NORC models can be strengthened by supplementing case management and social programming with basic needs support (e.g., food and housing assistance or counseling) as a standard NORC service. Case management, for example, might use a client navigation approach, focusing on residents with prior histories of high residential mobility to provide early intervention in cases of potential instability (e.g., indicated by missed rent). Case managers can also connect residents to public benefits and entitlement programs available to all New York City residents, providing a first-tier safety net including risk assessment, planning, and monitoring that may help older adults remain stably housed and navigate complex housing assistance programs, should they need them.

These findings also indicate that a significant number of participants report ever being evicted, moving twice or more in the past 5 years, or being concerned about housing costs. This is striking, considering participants were recruited from affordable housing that includes other older adult residents. Recruiting participants living in the same city but different boroughs (with different median area income levels) reveals that even in relatively affordable residential areas, such as the Bronx, where housing costs are slightly lower, cost burden may still be more prevalent, contributing to increased stress.

When housing costs do not fully reflect true area median level incomes, unemployment rates, or percent poverty, adults may be at greater risk of financial strain and instability, contributing to possible substance use as a coping strategy. For example, though residents living in Manhattan NORCs report higher incomes on average, potential rent burden due to a higher cost of living still poses risks, particularly among those who may move multiple times to maintain relative affordability. Disaggregating findings at the borough-level could facilitate addressing area-level costs of living and higher rates of substance use with community-based approaches and targeted resources; though generally, lowering housing costs for older adults requires coordinated efforts across city, regional, and federal levels.

### Housing instability as a correlate of health and substance use

This study supports prior research emphasizing links between housing instability and health in later life (36). Extending this research, we find that housing instability indicators are associated with higher rates of substance use, suggesting an underlying life concerns-coping mechanism, where housing costs may represent a significant source of strain and worry in older adults’ lives. We encourage additional research and longitudinal studies to explore this relationship since most substance use literature focuses on younger adults and children.

Our study fills an important gap by highlighting how housing insecurity correlates with substance use frequency among older adults; additional research can highlight how housing stress shapes negative behavioral outcomes that are relevant in older age. For example, cannabis and alcohol co-use is associated with a higher risk for falls, cognitive impairment, and medication interactions, all critical concerns for older adults aiming to age safely in place. Though these findings are promising, it is important to investigate reverse associations – whether lifetime substance use contributes to housing challenges and residential stability as an adult – using longitudinal study designs and tracking the impact of policy changes and use patterns over time.

### Limitations

Our study is not without important limitations. First, the sample population includes a subsample of older adults with a history of cannabis use; therefore, our results are not representative of all NORC residents living in New York City. Additionally, the subsample is relatively small and limited to mostly lower-income adults already living in NORCs, which may underestimate housing instability for more precariously housed older adults. Our small sample size also limits statistical power. For example, the majority of our sample earned less than $60k/year, and despite the importance of including lower-income samples for analysis, the small size of adults earning higher than $60k/year impedes the ability to examine specific income effects for higher-earning subgroups. As a pilot study, our findings are based on a convenience sample and we are unable to make broad claims about all older adults living in NORCS. However, the goal was to examine trends in a community-based sample of older adults with characteristics (e.g., a history of cannabis use) that may put them at increased risk for negative consequences. Second, though we recruited from NORC sites, we cannot verify that all our participants were NORC residents which may limit the scope of our findings. Our data, collected in 2022 during the later stages of the COVID-19 pandemic, also limits generalizability to other time periods and cities. Similarly, given that lifetime cannabis use was a criterion for eligibility, these findings may not apply broadly to all older adults. Finally, our two measures – concerns about housing costs and moving more than twice in 5 years – were treated as dichotomous measures to maximize power; however, we recognize that these decisions may have resulted in lost information.

As with any cross-sectional study, longitudinal research is needed to explore whether housing instability precedes or follows increased substance use. To explore whether substance use functions as a possible coping mechanism to manage housing-related stress requires a longitudinal approach.

Future research should also explore the impact of social isolation, full residential histories (e.g., evictions), and stressful life events (e.g., trauma), which may mediate the relationship between housing stress and substance use.

## Conclusion

Given that greater concern about paying for housing costs and frequent mobility are associated with poorer health and higher odds of tobacco and substance use, it is important to evaluate differences and variation across NORCs (in and across other cities) to understand and identify specific health risks related to substance use and other health behaviors among older adults. In less-resourced neighborhoods and boroughs, for example, living in a NORC may not fully mitigate housing costs or concerns, since poverty and unemployment rates are likely to be higher. NORC programs may consider implementing on-site case management, revising any existing case management structure to include client navigation and support specifically for basic needs, strengthening rent protections for adults over age 50, and supplementing referral services to include housing support and eviction defense agencies to help mitigate potential housing loss for residents. Other alternatives include identifying other tenants as navigators who might serve as a conduit (i.e., neighbors helping neighbors) for residents expressing financial difficulties and/or substance use-related harms, by offering support and connection to specific city resources for. Whether formal or informal, navigators can play a critical role in managing resident life in NORCs, providing intensive support, connecting residents with needed services, and helping them navigate complex systems (24, 35). Finally, substance-use counseling, smoking-cessation programs, and education incentives tailored specifically for older adults can be made available as part of NORC programming.

This study highlights the importance of context-specific research focused on housing affordability and stability for older adults, particularly in cities where housing costs are high and worries about increasing costs may be acute. Importantly, even within NORC-defined areas where services are designed to promote stability and aging-in-place, unmet housing needs remain a significant risk factor for poor health outcomes and substance use. Future research might recognize that instability can exist within NORCs; therefore, aging agencies and harm reduction programs serving older populations should prioritize those with housing insecurity. Understanding neighborhood contexts is an important priority for health prevention and aging-in-place research.

## Data Availability

The original contributions presented in the study are included in the article/supplementary material, further inquiries can be directed to the corresponding author.

## Footnotes

1
nyc.gov
